# Impact of a Medical Career on Reproductive Planning and Knowledge of Assisted Reproductive Technology Among Brazilian Women

**DOI:** 10.5935/1518-0557.20260027

**Published:** 2026

**Authors:** Kadija Rahal Chrisostomo, Ana Paula Muller Penachio, Camila de Angelis Bazzaneze, Najila de Marco Sandrin, Renato Nisihara

**Affiliations:** 1 Department of Gynecology and Obstetrics, Federal University of Paraná, Curitiba, PR, Brazil; 2 Department of Medicine, Positivo University, Curitiba, PR, Brazil; 3 Service of Gynecology and Obstetrics, Ipiranga State Hospital, São Paulo, SP, Brazil

**Keywords:** gynecology, fertility, reproductive techniques, assisted reproductive technologies, family planning

## Abstract

**Objective:**

To determine whether women in the medical field have different reproductive plans compared to women in other areas of higher education. Also, to assess their knowledge of assisted reproductive technologies (ART) and their interest in oocyte cryopreservation.

**Methods:**

This cross-sectional, analytical study was conducted using an anonymous online questionnaire administered between November and December 2024. The study included Brazilian women who were nulliparous, had higher education, and were divided into two groups: the study group (medical students and physicians) and the comparison group (women from other higher education fields). The questionnaire covered sociodemographic data, lifestyle habits, professional trajectories, affective relationships, reproductive intentions, and knowledge about ART.

**Results:**

A total of 905 women were included. The groups they were divided into had a similar age distribution. Most participants were aged between 18 and 29 years. The study group (n=490) reported higher income, greater levels of physical activity, and less smoking (*p*<0.0001). The impact of workload on relationship quality, academic plans, partner preferences, and perceived professional impact was more significant in the study group compared with controls (*p*<0.0001). They more frequently cited the demands of the medical profession as detrimental to their relationships. A higher proportion of women in this group planned to conceive later in life (61.8% vs. 34.1%; *p*<0.0001). Knowledge and acceptance of ART, especially oocyte cryopreservation, were significantly higher, with over 58% considering future use.

**Conclusions:**

A medical career greatly affects women’s reproductive choices, resulting in delayed motherhood despite strong reproductive desires. Providing early access to fertility education and ART options could be a strategy to promote reproductive autonomy among women in medicine.

## INTRODUCTION

In recent decades, women’s participation in Brazil’s formal workforce increased from 26.6% in 1980 to 53.3% in 2022 (IBGE, 2007; IBGE, 2024). This shift has transformed gender roles, redistributed time between work and family responsibilities, and influenced reproductive behavior. The average age at which women plan to have children has also risen: in 2000, 9.1% of pregnant women were over 35, and by 2020, this percentage grew to 16.5% (DataSUS, 2020). This trend is also reflected in medicine. In 1980, women made up 23.5% of physicians in Brazil (Scheffer, 2018), but by 2024, this percentage had increased to 49.9%. Notably, women now compose 60.5% of physicians aged 29 or younger (CFM, 2024). This demographic change has direct effects on fertility, reproductive planning, and awareness of assisted reproductive technologies (ART) among female physicians.

The proportion of women leaving the medical field at different training stages is substantially higher than that of men (Salem *et al*., 2022; Steiner-Hofbauer *et al*., 2023). As early as 2012, research indicated that physicians tend to have children later in life, with this delay being significantly more pronounced among women (Goldacre *et al*., 2012). One study on pregnancy decisions among female medical residents found that about 61% postponed pregnancy, mainly due to demanding work schedules (93%) and financial concerns (46%). Yet, only 38% were satisfied with this choice (Stack *et al*., 2020). Other studies also show a decreasing willingness among medical students to sacrifice quality of life for professional demands (Shifflette *et al*., 2018).

The extended time needed to finish medical education and achieve professional and financial stability-often followed by years of preparing for entrance exams and residency-also leads to delayed motherhood. Lack of structural and emotional support in the workplace also contributes to this delay (Theodosiou, 2013). Although some countries have implemented policies that support women in managing pregnancy and postpartum without harming their career progress (Salem *et al*., 2022), such measures are still largely unavailable to most female physicians in Brazil. Current evidence shows no clear agreement on how well women in the medical field understand the effects of age on fertility (Yu *et al*., 2016; Roberts *et al*., 2020; Alfaraj *et al*., 2019). However, many women who are aware of these effects say they wish they had learned about them earlier (Smith *et al*., 2022). In this context, assisted reproductive technologies (ART) can serve as important tools for reproductive planning among female physicians (Varlas *et al*., 2021). This study therefore aimed to determine whether being a woman in the medical field is linked to different reproductive planning patterns compared to women with similar education levels in other fields. It also examined the preferred age for pregnancy, the level of knowledge and interest in oocyte cryopreservation and assisted reproductive techniques, and how professional life affects the personal and family lives of the women surveyed.

## MATERIAL AND METHODS

### Ethical aspects

The project was approved under certificate 6.208.097 by the Research Ethics Committee at Positivo University. An anonymous, voluntary online survey was conducted from November to December 2024.

### Participants

The questionnaire was independently created by the authors and distributed as an online survey on the Google Forms® platform. Participant recruitment took place through digital channels, including social media platforms like Instagram and Facebook profiles of the researchers, groups of medical students, and personal blogs within Brazil. The survey link was also shared via WhatsApp groups. All responses were collected anonymously, and no identifying information was gathered from participants at any point during the study.

The respondents were split into groups:

***Study group:*** Female medical students or physicians were invited to participate. Inclusion criteria were: Brazilian women who were nulliparous and (i) currently enrolled in medical school (basic, clinical, or internship cycles), (ii) practicing physicians, or (iii) graduates who had studied or worked in Brazil; all provided consent by accepting the Informed Consent Form (ICF). Exclusion criterion: questionnaire responses deemed incomplete.

***Comparison group:*** Inclusion criteria consisted of Brazilian women who were nulliparous and either (i) students enrolled in non-medical undergraduate programs or (ii) graduates from non-medical higher education fields; all provided consent by signing the ICF. Exclusion criterion: incomplete questionnaire responses.

### Questionnaires

A review of the literature did not identify any validated instruments specifically related to the study topic. Therefore, the authors created a questionnaire based on existing scientific evidence and consultations with women-both physicians and non-physicians-who are involved in higher education. The questionnaire was then pilot-tested with 20 volunteers to evaluate clarity and comprehension. Participants gave feedback on questions that were unclear or difficult to understand, which helped improve the instrument before its use.

Two questionnaires were used for data collection: one for the study group and another for the comparison group, with some questions different for the study group (medical population) due to the length of their degree and their subsequent choice of specialty.

The questionnaire consisted of the following sections:

***Section 1 - Sociodemographic profile and lifestyle habits:*** age, ethnicity, height, weight, region and city of residence, gender identity, sexual orientation, monthly household or personal income, presence of comorbidities, physical activity levels using the International Physical Activity Questionnaire (IPAQ), use of anabolic steroids, smoking (assessed by the Fagerström Test), use of illicit substances, and alcohol consumption assessed with the CAGE questionnaire (Cut down, Annoyed by criticism, Guilty, Eye-opener)-a brief and validated screening tool to identify potential alcohol use disorders-were reviewed.

***Section 2 - Academic/Professional Life:*** Questions include relocation for academic reasons, age when starting college, current academic semester or years since graduation, area of professional interest or current career, current work location, plans to return to hometown, and key factors in choosing a professional specialty.

***Section 3 - Relationships:*** the presence of a stable relationship, whether personal plans have shifted due to a relationship, how workload affects relationships, relationship preferences, the impact of professional life on romantic relationships, the perceived link between academic or professional success and romantic life, whether socioeconomic status influences relationships, and if a romantic partner has influenced the choice of a specialty or career.

***Section 4 - Use of Contraceptives and Reproductive Planning: includes*** questions about contraceptive use, intentions to have children, the desired number of children and preferred age for having them, reasons for timing, key conditions considered before having children, and how professional background influences reproductive goals. It also covers causes of female infertility, awareness of and attitudes toward ART, whether ART is viewed as a feasible option, related costs, and views on adoption.

### Statistical analysis

After data collection, statistical analyses were conducted using SPSS version 17.0. To assess the normality of continuous variables, the Kolmogorov-Smirnov and Shapiro-Wilk tests were used. Continuous data are presented as medians with interquartile ranges (IQR) and compared with the non-parametric Mann-Whitney U test. Categorical variables are shown as proportions and analyzed using either Fisher’s exact test or the Chi-square test, depending on data distribution. A *p*-value less than 0.05 indicates statistical significance.

## RESULTS

A total of 905 women took part in the study, including 490 respondents from the medical group (students and physicians) and 415 women with higher education (either enrolled in or graduated from non-medical fields). No responses needed to be excluded.

Table 1 shows the sociodemographic characteristics of the study population. Age distribution was similar across both groups, with most participants (77.9% and 81.4%) aged between 18 and 29 years. The proportion of Afro-descendant individuals was significantly higher among non-medical women (7.5% *vs.* 17.5%; *p*<0.0001). In terms of sexual orientation, more bisexual participants were found in the non-medical group (13.8% *vs.* 27.9%; *p*<0.0001). Participants in the medical group reported significantly higher income levels (*p*<0.0001). Regarding lifestyle habits, the medical group reported higher levels of physical activity (29.1% *vs*. 19.2%) and a lower proportion of smokers (96.1% *vs.* 89.1%; *p*<0.0001). The non-medical group reported lower illicit substance use (*p*=0.022).

**Table 1:** Socio-demographic characteristics of the study population

	Medicine N=490 %	Other Professions N=415 %	*p* value^*^
Age (years) 18-24 25-29 30-39 ≥40	77.9 17.9 3.8 0.2	81.4 13.2 2.9 1.4	0.32
Ethnicity White Afro-descendent Other	84.2 7.5 8.2	72.7 17.5 9.6	<0.0001 <0.0001
Sexual orientation Heterosexual Homosexual Bisexual Other	80.8 3.6 13.8 1.6	65.0 4.3 27.9 2.6	<0.0001 0.78 <0.0001
Household income (monthly in minimum wages) ≤ 2 3-5 6-10 >10	15.9 24 25.3 34.6	45 33 12.7 9.1	<0.0001 0.62 <0.0001 <0.0001
Physical activity (IPAC) Very active Active Insufficiently active Sedentary	29.1 36.7 13.2 20.8	19.2 26.9 17.8 35.9	0.0004 0.00036 0.42 <0.0001
Smoking cigarettes/day I do not smoke <10 ≥10	96.1 2.8 1.0	89.1 9.1 1.6	<0.0001
Alcohol use (CAGE) I do not drink 1x/month 2-3x/month 1x/week 2x/week 3-4x/week Daily use	26.1 22.4 32.0 12.4 5.7 1.2 0	32 19.2 32 8.6 2.8 0 0	0.061
Illicit substance use Yes	24.7	23.4	0.022

IPAQ: International Physical Activity Questionnaire; CAGE: Cut down, Annoyed by criticism, Guilty e Eye-opener.

* Chi-Square test.

Table 2 presents data about the academic and professional lives of the study groups. There are no significant differences between the groups. Most participants began college between ages 18 and 22. Only 7.5% of the medical group had graduated, compared to 15.6% in the other profession.

**Table 2:** Data about the academic/professional life of the study population

	Medicine N=490 %	Other Professions N=415 %	*p* value
Age when you started going to college 16 - 18 19 - 22 23 - 25 26 - 29 30 or more	34.9 51.1 9.3 2 2	57.7 35.5 2.4 2.1 1.4	0.48
Semester at college 1 or 2 3 or 4 5 or 6 7 or 8 9 or 10 11 or 12 I have graduated from college	27 19.3 23.1 13.2 5.1 4.9 7.5	14.9 21.7 21.3 14.4 12 0 15.6	0.14
Years since graduation 0 to 2 years 3 to 5 years 6 to 10 years I have not graduated from college yet	6.5 1.4 0.2 91.9	9.2 5.0 3.4 83.2	0.21

* Chi-Square test.

Data on relationship aspects are shown in Table 3. Workload significantly influenced relationship quality among participants in the medical group (33.4% *vs.* 22.6%; *p*<0.0001). Women in this group more often reported changing their academic plans because of a relationship (38.3% *vs*. 29.3%), preferred partners from the same profession (31.8% *vs.* 7.2%), and believed that their profession affected their relationships, all at significantly higher rates than the comparison group. Additionally, they more frequently reported that their profession made affective relationships more challenging, that professional success conflicted with their romantic life, and that it prevented them from forming emotional connections more often than women outside the medical field.

**Table 3:** Relationships in the study population

	Medicine N=490 %	Other Professions N=415 %	*p* value^*^
Does work interfere with the quality of your relationships? Family Friends Romantic partners Does not interfere	45.3 54 42 25.1	37.8 36.8 35.1 40.9	0.32 <0.0001 0.0186 <0.0001
Steady partner Yes	64	60.9	0.25
Changing academic plans for a relationship Yes	38.3	29.3	0.0044
Preference in partners profession Same profession Indifferent	31.8 46.1	7.2 63.1	<0.0001 <0.0001
Impact of work on affective relationships Big Small None	32.8 55.5 11.6	22.4 57.8 19.7	0.0037 0.50 0.00049
Work as an obstacle in affective relationships No negative impact Workload produces negative impacts	24.4 33.4	36.1 22.6	0.00015 0.00013
Professional success hinders affective relationships Yes	42.4	33.4	<0.0001
Work is an obstacle to developing affective relationships Yes	37.7	19.2	<0.0001

* Chi-Square test.

Table 4 presents data on contraceptive methods and knowledge about infertility in the studied groups. Women in the medical group reported more frequent use of contraceptive methods overall (82.2% *vs.* 72.0%). The oral contraceptive pill was the most commonly used method in both groups, with no significant difference between them. However, the use of hormonal intrauterine devices (IUDs) was significantly higher among women in the medical group. A larger proportion of participants in the non-medical group reported not planning to have children (11.6% *vs*. 26.9%; *p*<0.0001). Women in the medical group expressed a desire to have more children, but at an older age, primarily due to career stability and the perceived impact of their profession on childbirth timing. As expected, knowledge about assisted reproductive technologies (ART) was higher among women in the medical group. Additionally, over 58% of them considered the possibility of undergoing ART if needed, a rate significantly higher than in the comparison group.

**Table 4:** Data on contraceptives and knowledge of infertility among the studied groups

	Medicine N=490 %	Other professions N=415 %	*p* value^*^
Use of contraceptives Yes No	82.2 17.3	72.0 24.8	0.00026 0.0058
Contraceptive method in use Condom Hormonal IUD Copper IUD Pill Injectable Contraceptive implant	40.8 18.1 .4 42.2 1.8 5.5	45.7 7.2 5.0 39 2.6 0.9	0.76 <0.0001 0.62 0.14 <0.0001 <0.0001
How many children do you intend to have? None 1 2 ≥3	11.6 12.6 57.7 17.9	26.9 19.5 39.2 14.2	<0.0001 <0.0001 <0.0001 <0.0001
At what age would you like to have children? 21-25 years 26-30 years 31-35 years ≥ 36 years	1.6 25.7 56.1 5.7	4.8 37.1 29.8 3.3	<0.0001 <0.0001 <0.0001 <0.0001
Rationale for choosing the age to have children I want to have children as soon as possible I must have a stable career first	11 70.6	5.3 63.1	0.0012 0.0014
Does your profession affect the age at which you intend to have children? Yes	93.0	55.6	<0.0001
Knowledge about the relationship between age and fertility Yes	98.3	83.3	<0.0001
Knowledge about causes of female infertility Yes	76.7	44.5	<0.0001
Did you select your field of specialty based on your wish to have children? Yes	53.6	27.9	<0.0001
Knowledge about assisted reproduction methods Yes	90.2	81.4	<0.0001
Have you considered assisted reproduction? Yes	58.5	40.4	<0.0001
Interest in freezing eggs Yes	56.3	33.4	<0.0001
After considering the cost of freezing eggs, are you still interested in doing it? Yes	49.1	14.9	<0.0001
Interest in adoption Yes	50.6	53.0	0.48

IUD: intrauterine device.

* Chi-Square test.

## DISCUSSION

The proportion of women in Brazilian medicine has steadily increased and is expected to surpass that of men. Women now make up 50.9% of healthcare professionals, with projections reaching 55.7% by 2035 (Scheffer, 2025). However, research on reproductive planning among Brazilian female doctors remains limited. This study offers robust data on how professional paths and career decisions influence reproductive choices. Concerning assisted reproduction, results show that delaying motherhood is a real trend. Despite the troubling link between having a medical career and delayed motherhood, many women report a lack of information and support. These findings emphasize the need for strategies to improve access and encourage discussions about fertility preservation.

Regarding sociodemographic distribution, women in both groups had similar ages, with a higher proportion of Afro-descendants and lower household incomes in the non-medical group. These findings align with data from Brazilian medical training programs, especially in private universities. The presence of Black individuals in medical programs remains low, with 2.8% Black and 19.1% mixed race among graduates; the number is even lower for Black women (INEP, 2024). Regarding lifestyle habits, participants in the medical group engage in more physical activity and smoke less than the comparison group. However, they use more illicit substances. It is possible that greater knowledge about the benefits of physical activity and the harms of smoking are more widely discussed in medical programs, contributing to these figures. On the other hand, there is also greater contact with illicit substances. Most respondents in both groups were undergraduates, with participants evenly distributed across study years. About half of the medical group started between ages 19 and 22. Given the course length and pursuit of residency, graduation likely occurs around age 30. These findings align with other surveys and highlight concerns about family planning (INEP, 2024).

When asked about the impact on workload, two-thirds of medical students reported negative effects on their relationships with family, friends, and romantic partners, a proportion significantly higher than that of the comparison group. Many expressed they would alter their academic plans for a relationship and preferred partners in the same field, recognizing that medical training interferes with personal bonds. This preference may reflect a desire for mutual understanding and support. Participants also noted that professional success and pursuing medicine could decrease the approachability and interest of potential partners. Overall, the findings suggest that choosing a medical career early influences students’ personal and social lives, affecting reproductive planning as many postpone family formation to prioritize professional development. Some even reconsider academic or career paths due to relationships, highlighting the tension between aspirations and emotional needs. While studies have examined the effects of a medical career on relationships and psychological outcomes (Trockel *et al*., 2024; Karakash *et al*., 2019; Gold *et al*., 2025), analyses from a female perspective-particularly regarding reproductive planning-remain limited, especially in the Brazilian context.

Regarding contraceptive use, a significantly higher number of women in the medical group reported using contraception, with more frequent use of hormonal IUDs and hormonal implants. This may be linked to higher household income and greater awareness of these methods within this group. It was also observed that the proportion of women who do not want children is significantly higher in the non-medical group. Additionally, women in the medical group showed a greater intention to have more children, indicating a stronger reproductive desire, a finding that is unprecedented in the literature.

An expected and relevant finding was that the planned age for pregnancy was significantly higher among participants in the medical group, which can be explained by their longer training period compared to the control group. For more than 90% of participants in the medical group, their professional choices influence the age at which they decide to have children. This trend may be linked to the stigma that pregnancy and motherhood still face within the medical field. Ongoing obstacles include heavy workloads and gender bias (Casilla-Lennon *et al*., 2022). It is also important to highlight that delaying pregnancy may be associated with infertility, increased miscarriage rates, and other pregnancy complications related to advanced maternal age (Bakkensen *et al*., 2023; Cusimano *et al*., 2021; Simpson *et al*., 2021).

As expected, medical participants had greater knowledge of reproductive medicine. More than half consider this method a future alternative and express interest in freezing eggs, even knowing the costs. In this regard, American studies have already demonstrated the economic impact on the lives of female physicians due to the investment involved in the assisted reproduction process (Veade *et al*., 2023a). It is also important to highlight the difference in the pursuit of assisted reproduction techniques, due to increased infertility, among female physicians in surgical careers compared to those in clinical careers (Phillips *et al*., 2014). Furthermore, scientific evidence shows that providing support for fertility treatments helps retain women in medicine in the long run, while also reducing burnout rates and promoting personal and professional growth (Veade *et al*., 2023b). No difference was observed in the desire to adopt children among the groups studied.

This study has some limitations due to its cross-sectional design and reliance on an online questionnaire. However, this approach reduces the embarrassment of answering personal questions and strengthened the study, which included a large number of participants-probably because the women were interested in the topic and felt comfortable sharing their opinions. Unfortunately, the number of graduated women in both groups was smaller than ideal, which limited the comparisons.

The data from our study can support further research into interventions that help healthcare and medical higher education institutions minimize these impacts. It can also evaluate the effectiveness and best ways to implement psychological and social support programs for women in medicine, with a positive impact on their quality of life. Undoubtedly, discussing fertility preservation in this group is justified.

## CONCLUSION

Brazilian women in medicine showed different reproductive planning compared to women in other fields, marked by later childbearing but higher reproductive desire and ART awareness. This study also emphasizes the complexity of women physicians’ experiences, showing how academic and professional pressures impact personal relationships and directly influence reproductive planning.
